# High Incidence, Reinfections, and Active Syphilis in Populations Attending a Specialized HIV Clinic in Mexico, a Dynamic Cohort Study

**DOI:** 10.1007/s10508-022-02433-1

**Published:** 2022-09-29

**Authors:** Omar David Tumalán-Gil, Verónica Ruiz-González, Santa García-Cisneros, Andrea González-Rodríguez, Antonia Herrera-Ortiz, Maria Olamendi-Portugal, Miguel Angel Sánchez-Alemán

**Affiliations:** 1 Centro de Investigación sobre Enfermedades Infecciosas, Instituto Nacional de Salud Pública, Universidad No. 655 Colonia Santa María Ahuacatitlán, Cerrada Los Pinos y Caminera, C.P. 62100 Cuernavaca, Morelos México; 2 Clínica Especializada Condesa, Cd. de México, México; 3 Coordinación Programa VIH/SIDA, Cd de México, Mexico

**Keywords:** Syphilis, Syphilis serodiagnosis, Cohort studies, Incidence, HIV, Sexually transmitted infections

## Abstract

Syphilis, a sexually transmitted infection, has reemerged in many vulnerable groups around the world. The objective of the current study was to determine the prevalence and incidence of syphilis among people who attended a specialized HIV clinic in Mexico from 2011 to 2015. Databases from the laboratory were analyzed, and the following four groups were formed: people seeking HIV-1 voluntary counseling and testing (VCT), people in prison (PPr), people living with HIV (PLWH), and patients from primary care clinics (others). The diagnosis of syphilis was made using the reverse algorithm; antibody titers were examined to determine the stage of infection. Baseline data were analyzed and, with follow-up information, a retrospective dynamic cohort was formed. Factors associated with the seroprevalence of syphilis and active syphilis were evaluated by the chi-square test. Moreover, risk factors for the incidence of syphilis were described. A total of 81,863 baseline individuals were analyzed. The seroprevalence of syphilis was 9.9% in the VCT group, 8.2% in the PPr group, 37.0% in the PLWH group, and 8.7% in the others group; the prevalence of active syphilis was 1.7–13.1%. A total of 11,124 people were followed up. The incidence (cases per 100 person-years) was 3.5 among the VCT group, 16.0 among the PLWH group, and < 0.1 among both the PPr and others groups, respectively; moreover, the frequency of reinfections was 11.1–24.4%. The high prevalence and incidence of syphilis, active syphilis, and reinfections among men, transgender people, individuals aged 20–39 years, and people with a history of HIV or hepatitis B suggest that it is critical to improve prevention, diagnosis, and treatment measures to stop the reemergence of syphilis. There are also new factors such as methamphetamine use, group sex, or contacting partners over the internet that are associated with syphilis. In addition, HIV preexposure prophylaxis could contribute to the increased incidence of syphilis by providing false security in the prevention of STIs, thereby increasing risky sexual behaviors.

## Introduction

The World Health Organization (WHO) estimated an increase in the number of syphilis cases, from 5.6 million cases in 2012 to 6 million cases in 2016. Likewise, the prevalence of syphilis was 3.2% among female sex workers (FSW) and 6.0% among men who have sex with men (MSM). The age-standardized incidence rate was 178.48 per 100,000 people in 2019, with the Americas having the highest increase in syphilis incidence between 2010 and 2019. On the other hand, the WHO established the objective of reducing the incidence of syphilis by 90%, with a focus on priority groups, such as MSM, FSW, and intravenous drug users (IDU) (Du et al., 2022; Newman et al., 2015; WHO, 2018).

Syphilis is a sexually transmitted infection (STI) caused by the bacterium *Treponema pallidum*, which also causes congenital syphilis, and has increased in different parts of the world. Between 2013 and 2017, the number of cases per 100,000 inhabitants in the US increased from 5.5 to 9.5 (CDC, 2018). The European Economic Community reported an increase from 4.7 cases to 6.1 cases per 100,000 inhabitants between 2012 and 2016 (ECDC, 2018). In Mexico, an increase in syphilis was documented among young men, focused in Mexico City (Herrera-Ortiz et al., 2015), and it was also recently observed in the general population. Syphilis increased from 2.03 cases per 100,000 inhabitants in 2010 to 6.09 cases in 2019. Finally, the number of cases of congenital syphilis grew from 62 cases in 2010 to 372 cases in 2019 (García-Cisneros et al., 2021). Syphilis is important because of the number of cases and the higher risk of acquiring and transmitting HIV; syphilis increases the risk of contracting HIV by 2.5 times; moreover, coinfection with syphilis and HIV negatively modifies both diseases (Lynn et al., 2004). In the last decade, coinfection with syphilis/HIV among MSM has been higher than that among heterosexual men; it is possible that MSM living with HIV engage in risky sexual behavior (Roberts et al., 2016). The reemergence of syphilis is not only due to individual sexual behaviors, such as early age of sexual debut, high number of sexual partners, and nonuse of condoms (Conde-Gonzalez et al., 2007). There are also cultural, social, and structural factors, such as discrimination and stigma, which are common in Mexico due to the culture of machismo. There are also cultural, social, and structural factors, such as discrimination and stigma, which is common in Mexico due to the culture of machismo (Espinosa da Silva et al., 2020).

The Clinica Especializada Condesa (CEC) in Mexico City is the institution that serves the largest number of people seeking HIV testing, as well as people living with HIV (PLWH). The CEC also performs tests for other STIs, such as hepatitis B virus (HBV), hepatitis C virus (HCV), and syphilis. The objective of the current study was to determine the prevalence, incidence, and risk factors for syphilis among populations attending the CEC from 2011 to 2015.

## Method

A database was elaborated from the CEC with the variables, including year (2011–2015), internal number, population type, sex, age, HIV test, HBV test, HCV test, syphilis test, and syphilis titers. The study of STIs considers different vulnerable populations with particular characteristics for their prevention and treatment. In the present study, the population was stratified into four groups, according to the classification made by the CEC: (1) people seeking HIV-1 voluntary counseling and testing (VCT); (2) PLWH, who were people with HIV before syphilis testing, no social security and who were attending the CEC for HIV treatment; (3) people in prison (PPr); and (4) patients from primary clinics, people receiving postexposure prophylaxis for sexual violence and pregnant women and children at risk for syphilis (others). The age variable was stratified in decades. The CEC sends syphilis patients to their primary care clinics for treatment, and only PLWH without social security attend the CEC for their treatment.

In relation to the detection of HIV, a rapid test is performed, and if it is positive, it is confirmed with one of the following tests: Abbott Architect HIV Ag/Ab Combo, Abbott HIV-1 RNA RT-PCR or Orgenics Immunocomb II HIV 1 & 2 Combfirm. If the rapid test was negative, 4 samples were pooled and analyzed with the Abbott Architect HIV Ag/Ab Combo (Juárez-Figueroa et al., 2020). The reverse algorithm was used for the detection of syphilis a treponemal test was applied (rapid test or Abbott Architect TP), and if it was positive, the nontreponemal VDRL test was applied to determine the antibody titer. Some authors have mentioned that, in the absence of clinical data, the antibody titer can be used to determine the stage of syphilis, such as active syphilis (titer ≥ 1:8), latent syphilis (titer 1:1–1:4) and cured syphilis (VDRL negative, positive treponemal test) (Conde-González et al, 2007; Zoni et al., 2013). Additionally, Architect HBsAg tests were used for the detection of HBV antigen and Architect anti-HCV tests for the detection of antibodies against HCV. The inclusion criteria were to have all the demographic variables and laboratory results for HIV and syphilis. For the dynamic cohort, it had two measurements.

The present study was approved by the Ethics Committee of the National Institute of Public Health of Mexico. The research was carried out in accordance with national guidelines and the Declaration of Helsinki. Everyone who attends the Clinica Especializada Condesa is asked to sign an informed consent letter. The database had identification keys, and we did not have access to the names of the participants.

From the baseline information, frequency analysis stratified by population group was performed, and chi-square (*χ*^2^) tests were used to evaluate the differences. Subsequently, a bivariate analysis with *χ*^2^ was performed to determine associated variables, regarding both the seroprevalence of syphilis and the presence of active syphilis. The *χ*^2^ test for trend was used for the year and age variables, considering the four population groups.

A retrospective dynamic cohort was elaborated with individuals who had two syphilis tests with at least four months between measurements, and incidence densities were calculated using person-years (py). During follow-up, syphilis was considered cured if the antibody titer decreased until negative or if the antibody concentration decreased four times or more. Syphilis reinfection was defined when it was cured at baseline and the titer was ≥ 1:1 during follow-up or if the antibody titer increased four times or more. Persistent infection was considered if the antibody titer remained similar (± 2 times) (Workowski et al., 2015). Incidence rate ratios (IRRs) were calculated with 95% confidence intervals (CIs), and a cumulative incidence curve was constructed using the Kaplan–Meier method. A p value < 0.01 was considered statistically significant. Statistical analysis was performed using the SPSS 15.0 software.

## Results

### Baseline

A total of 129,613 blood/serum samples were processed in the CEC laboratory between 2011 and 2015. In 2011, there were 17,589 samples, and in 2015, there were 37,342 samples. The results for syphilis detection were from 95,732 samples, of which 81,857 had complete information; 78.1% were in the VCT group, 9.9% in the PPr group, 2.2% in the PLWH group, and 9.8% in the others group. Table 1 shows the demographic characteristics and laboratory results of the four analyzed populations. In all groups, there was an increase in the number of samples analyzed over the years, with the exception of PPr, which presented the greatest number of samples during 2012–2013. Men were the majority in all groups, from 63.3% in the others group to 85.5% in the PLWH group. The highest percentage of transgender people was in the PLWH group. The PLWH group had the highest average age (34.6 years), while the VCT group had the lowest average age (29.0 years). In all, 25% of individuals in the VCT group were HIV-positive, while 3.8% of individuals in the PPr group were HIV-positive. In the PLWH group, 4.9% of the individuals had HBV antigens, while 2.9% of the individuals had antibodies against HCV.

**Table 1:** Demographic and clinical characteristics in populations attending a specialized HIV clinic in Mexico

	VCT *N* = 63,916	PPr *N* = 8077	PLWH *N* = 1808	Others *N* = 8056	*P*
	% (*n*)	% (*n*)	%(*n*)	%(*n*)	
*Year*					
2011	8.9 (5683)	16.1 (1297)	18.8 (339)	13.3 (1068)	** < 0.001**
2012	19.3 (12,323)	25.3 (2045)	24.5 (443)	17.5 (1406)	
2013	21.9 (14,000)	22.3 (1800)	19.1 (345)	24.5 (1976)	
2014	23.4 (14,982)	17.3 (1396)	14.8 (268)	23.3 (1874)	
2015	26.5 (16,928)	19.1 (1539)	22.8 (413)	21.5 (1732)	
*Sex*					
Female	34.3 (21,946)	31.8 (2568)	11.9 (215)	36.6 (2945)	** < 0.001**
Male	64.3 (41,118)	67.2 (5427)	85.5 (1546)	63.3 (5100)	
Transsexual	1.3 (856)	1.0 (82)	2.6 (47)	0.1 (11)	
*Age (years)*					
≤ 9	1.1 (680)	−(0)	−(0)	7.9 (640)	** < 0.001**
10–19	11.7 (7460)	2.4 (192)	2.0 (36)	19.2 (1545)	
20–29	49.9 (31,896)	33.7 (2721)	32.2 (583)	28.7 (2311)	
30–39	22.0 (14,077)	37.9 (3061)	36.8 (666)	19.8 (1599)	
40–49	9.9 (6351)	18.8 (1521)	21.7 (393)	12.4 (1002)	
50–59	3.9 (2465)	5.9 (479)	6.1 (111)	6.8 (551)	
≥ 60	1.5 (987)	1.3 (103)	1.1 (19)	5.1 (408)	
*HIV test*					
Negative	81.4 (52,035)	91.0 (7353)	−(0)	86.0 (6930)	** < 0.001**
Positive	18.6 (11,865)	3.8 (308)	100 (1808)	7.2 (584)	
Not done	0.02 (16)	5.2 (416)	−(0)	6.7 (542)	
*HBV test*					
Negative	89.1 (56,966)	74.2 (5993)	77.3 (1397)	83.9 (6762)	** < 0.001**
Positive	0.9 (551)	0.9 (69)	4.9 (89)	0.6 (45)	
Not done	10.0 (6399)	24.9 (2015)	17.8 (322)	15.5 (1249)	
*HCV test*					
Negative	95.4 (60,995)	81.5 (6579)	81.7 (1477)	86.0 (6928)	** < 0.001**
Positive	0.9 (598)	3.1 (253)	2.9 (52)	1.4 (110)	
Not done	3.6 (2323)	15.4 (1245)	15.4 (279)	12.6 (1018)	

*VCT*: people seeking HIV-1 voluntary counseling and testing; *PPr*: people in prison; *PLWH*: people living with HIV; *Others*: patients from primary care clinics. Bold: *p* < 0.01 statistically significant. The frequencies for each population analyzed are shown (percentages vertically)

**Fig. 1:**
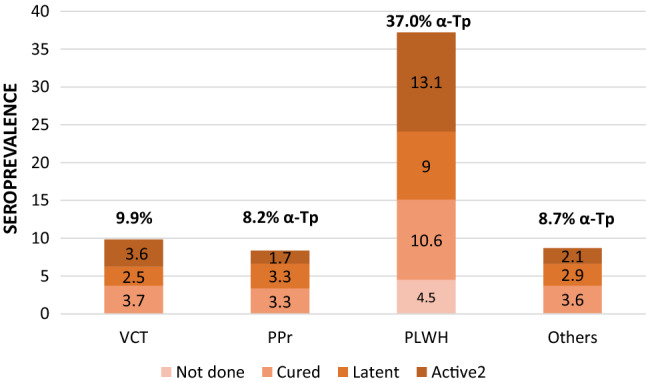
Seroprevalence of *Treponema pallidum* and active syphilis in populations attending a specialized HIV clinic in Mexico. The seroprevalence of syphilis was 9.9% (6339/63,916) among the people seeking HIV-1 voluntary counseling and testing (VCT) group, 8.2% (663/8077) among the people in prison (PPr) group, 37.0% (669/1808) among the people living with HIV (PLWH) group and 8.7% (701/8056) among the patients from primary care clinics (others) group. Antibody titers were not performed in 0.2% of the VCT group, in 0.04% of the PPr group, in 4.5% of the PLWH group and in 0.1% of the others group

The seroprevalence of syphilis was 8.2% (95% CI 7.6–8.8) among the PPr group, 8.7% (95% CI 8.1–9.3) among the others group, 9.9% (95% CI 9.7–10.1) among the VCT group, and 37.0% (95% CI 34.8–39.2) among the PLWH group. Figure 1 shows the seroprevalence of syphilis and active syphilis. Almost 40% of people with antibodies showed a cured infection, except among PLWH, in which the percentage was 32.4%. The VCT and PLWH populations had the highest seroprevalence of active syphilis, with 3.6% and 13.1%, respectively.

The seroprevalence of syphilis decreased over time in the VCT and PPr groups (*p* < 0.001). The transgender population had the highest seroprevalence; it was highest among the PPr group, followed by the PLWH group, with more than 40% in both cases (*p* < 0.001). At an older age, the seroprevalence of syphilis increased in the four analyzed groups (*p* < 0.001). The population of children aged 9 years or less presented the lowest prevalence of syphilis, with 0.7% and 0.9% in the VCT and others groups, respectively. Individuals with other STIs (HIV, HBV, or HCV) had a higher association with the seroprevalence of syphilis (*p* < 0.05) (Table 2, left).

**Table 2:** Seroprevalence of *T. pallidum* and active syphilis in populations attending a specialized HIV clinic in Mexico

	Seroprevalence T. *pallidum* %				Active syphilis			
	VCT	PPr	PLWH	Others	VCT	PPr	PLWH	Others
*Year*								
2011	13.2	9.8	37.5	2.0	4.7	1.8	4.7	2.0
2012	9.7	7.7	38.8	2.5	3.6	1.4	13.5	2.5
2013	9.5	9.2	33.0	2.0	3.4	1.8	15.7	2.0
2014	9.2	8.8	38.4	1.9	3.1	1.6	14.6	1.9
2015	9.9	5.9	37.0	2.4	3.8	1.9	16.2	2.4
p (Χ^2^ trend)	** < 0.001**	**0.006**	0.812	0.700	** < 0.001**	0.775	** < 0.001**	0.700
*Sex*								
Female	1.7	7.1	14.9	2.3	0.4	0.8	3.7	2.3
Male	14.1	8.2	39.9	2.1	5.3	1.9	14.2	2.1
transsexual	18.9	43.9	42.6	0.0	5.0	13.4	17.0	0.0
p (Χ^2^)	** < 0.001**	** < 0.001**	** < 0.001**	0.720	** < 0.001**	** < 0.001**	** < 0.001**	0.720
*Age (years)*								
≤ 9	0.7	–	–	0.9	0.0	–	–	0.9
10–19	3.0	5.7	13.9	1.6	1.8	2.6	13.9	1.6
20–29	9.0	7.5	29.0	2.8	4.0	2.1	14.8	2.8
30–39	13.5	8.1	39.9	1.6	4.6	1.5	13.5	1.6
40–49	12.9	8.4	42.0	2.6	3.1	1.1	11.5	2.6
50–59	13.1	10.2	48.6	2.5	2.1	1.5	9.0	2.5
≥ 60	18.2	22.3	52.6	2.7	1.4	2.9	0.0	2.7
p (Χ^2^ trend)	** < 0.001**	** < 0.001**	** < 0.001**	0.021	** < 0.001**	0.217	0.223	0.021
*HIV test*								
Negative	5.1	6.5	–	0.8	1.8	1.2	–	0.8
Positive	30.8	29.5	37.0	7.9	11.4	10.7	13.1	7.9
p (Χ^2^)	** < 0.001**	** < 0.001**	**–**	** < 0.001**	** < 0.001**	** < 0.001**	**–**	** < 0.001**
*HBV test*								
Negative	9.9	7.9	34.0	1.3	3.6	1.8	11.3	1.3
Positive	41.9	34.8	49.4	13.3	17.2	10.1	21.3	13.3
p (Χ^2^)	** < 0.001**	** < 0.001**	** < 0.001**	** < 0.001**	** < 0.001**	** < 0.001**	** < 0.001**	** < 0.001**
*HCV test*								
Negative	9.9	7.8	32.8	1.4	3.6	1.9	11.5	1.4
Positive	26.6	12.3	53.8	3.6	9.5	1.6	19.2	3.6
p (Χ^2^)	** < 0.001**	**0.003**	** < 0.001**	** < 0.001**	** < 0.001**	0.030	** < 0.001**	** < 0.001**

Seroprevalence of antibodies against *T. pallidum*. *VCT*: people seeking HIV-1 voluntary counseling and testing; *PPr*: people in prision; *PLWH*: people living with HIV; *Others*: patients from others clinics. Active syphilis, titters ≥ 1:8 in non-treponemic tests. Bold: *p* < 0.01 statistically significant

When analyzing the frequency of active syphilis, there was a decrease during the study period in the VCT group. In contrast, active syphilis increased among the PLWH group during the analyzed period. Women presented with the lowest proportion of active syphilis in the four groups; the highest frequency of active syphilis was in the transgender and men living with HIV populations at 17.0% and 14.2%, respectively (*p* < 0.001). People aged 20–29 years and 30–39 years had the highest frequency of active syphilis in the VCT group. A history of STIs was associated with a higher frequency of active syphilis in the entire population (Table 2, right).

### Dynamic Retrospective Cohort

**Fig. 2:**
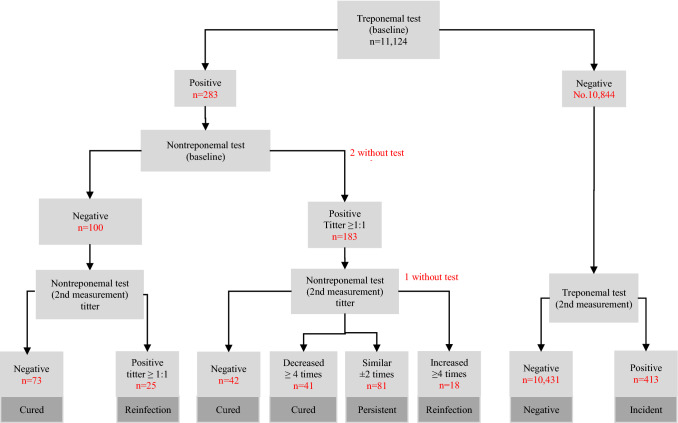
Retrospective dynamic cohort in populations attending a specialized HIV clinic in Mexico

There were samples from 11,124 individuals with a baseline syphilis outcome and at least one additional visit to the CEC, and the median time between visits was 10.6 months (interquartile interval 6.0–18.0). Figure 2 shows that, from baseline, 10,844 people were negative for the treponemal test, and 283 were positive for the treponemal test. During follow-up of negative samples, 413 positive samples were detected in the second measurement. Considering the nontreponemal test at baseline and second measurement, 59.6% had a cured infection, 13.6% had reinfection, and 26.8% had persistent infection among the VCT group; 29.6% had a cured infection, 11.1% reinfection, and 59.3% persistent infection among the PPr group; 56.1% had cured infection, 24.4% reinfection, and 19.5% persistent infection among the PLWH group; and 50.0% had cured infection, 21.4% reinfection and 28.6% persistent infection in the others group. Considering all groups, 49.4% of persons with a persistent syphilis infection had twelve months or more between the two measurements (treatment failure). Among people without HIV, this percentage was 41.9% (26/62), and among PLWH, it was 73.7% (14/19), with the difference being statistically significant (*p* = 0.019).

**Fig. 3:**
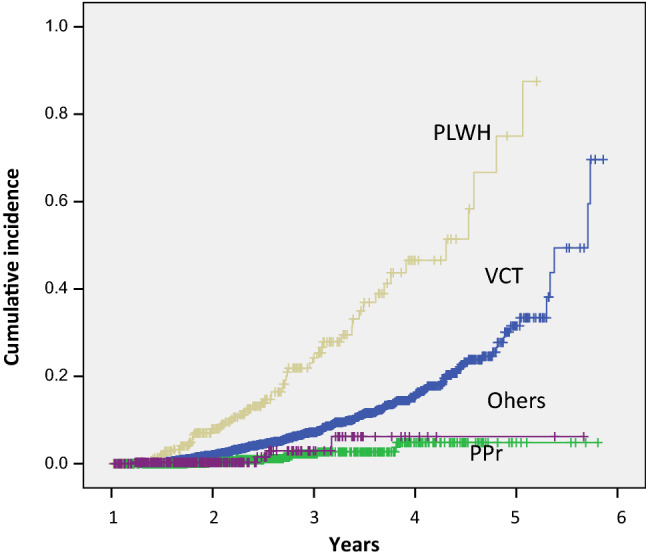
Cumulative incidence of *Treponema pallidum* in populations attending a specialized HIV clinic in Mexico. VCT: People seeking HIV-1 voluntary counseling and testing; PPr: people in prison; PLWH: people living with HIV; Others: patients from primary care clinics

The highest incidence of syphilis was found in the PLWH group with 16.0 cases per 100 py (95% CI 12.6–20.3), followed by the VCT population with 3.5 cases per 100 py (95% CI 3.1–3.8), the PPr group with 0.95 cases per 100 py (95% CI 0.5–1.7), and finally others with 0.86 cases per 100 py (95% CI 0.3–2.6). Among the PLWH group, at 39.67 months (95% CI 30.91–48.41), 50% of the population had acquired syphilis, which is a value lower than that of the VCT group (52.5 months; 95% CI 48.51–56.5). This difference was statistically significant (*p* < 0.001; log rank) (Fig. 3). For the other groups, it was impossible to calculate the infection time due to the sample size and the low proportion of positive cases.

Table 3 shows the IRRs for syphilis. Notably, the incidence increased in relation to the calendar year, but this change was not statistically significant. Men and transgender individuals had an incidence of 4.6 cases per 100 py and had a 9-times greater risk of having syphilis than women. The 20–29- and 30–39-year age groups had the highest incidence of *T. pallidum* infection, with 3.9 and 3.4 cases per 100 py, respectively. These groups also had twice the risk of infection compared to individuals over 50 years old. People with a history of HIV infection had an incidence of 12.2 cases per 100 py, those with a history of HBV infection had an incidence of 11.4 cases, and individuals with HCV infection had an incidence of 1.7 cases per 100 py.

**Table 3:** Incidence and risk factors for *T. pallidum* infection in populations attending a specialized HIV clinic in Mexico

	Cases/years follow-up	Incidence per 100 py*	IRR**
*Year*			
2015	10/309	3.23	1.03 (0.47–2.02)
2014	70/1867	3.75	1.20 (0.84–1.70
2013	126/3288	3.83	1.22 (0.90–1.67)
2012	140/4238	3.30	1.05 (0.78–1.43)
2011	67/2137	3.14	1.0
*Sex*			
Male	386/8411	4.59	**8.68 (5.35–15.06)**
Transgender	10/213	4.69	**8.88 (3.64–20.54)**
Female	17/3217	0.53	1.0
*Age (years)*			
≤ 9	0/51	–	
10–19	31/956	3.24	1.82 (0.79–4.91)
20–29	244/6267	3.89	**2.19 (1.05–5.51)**
30–39	99/2951	3.35	1.89 (0.88–4.82)
40–49	31/1120	2.77	1.56 (0.67–4.19)
≥ 50	7/394	1.78	1
*HIV baseline*			
Positive	113/923	12.24	**4.42 (3.53–5.51)**
Negative	298/10,767	2.77	1.0
*HBV baseline*			
Positive	9/78	11.54	**3.21 (1.46–6.17)**
Negative	343/9,554	3.59	1.0
*HCV baseline*			
Positive	2/114	1.75	0.5 (0.1–1.82)
Negative	386/11,009	3.51	1.0

*py: persons-years of follow-up; *bold*: statistically significant. **IRR**:** Incidence rate ratio

## Discussion

The prevalence of syphilis in the groups that attended the CEC was high (from 8.2% among the PPr group to 37.0% among the PLWH group) compared to other vulnerable populations, such as FSWs (14.2%) (Patterson et al., 2008), PPr (2.0%) (Bautista-Arredondo et al., 2015), and MSM (9.5%, 14.8%, and 30% in 2014, 2016, and 2017, respectively) (SS, 2018). The higher prevalence of syphilis among the PPr group in the current study may be because the study was carried out in later years and the diagnostic methods were considered. In the current study, even cured syphilis was considered, unlike the 2010 study, which did not consider it (Bautista-Arredondo et al., 2015). Likewise, the prevalence detected at the CEC is higher than in certain groups, such as pregnant women (0.26%), blood donors (1.4%), and the general population (3.1%) (Bautista-Arredondo et al., 2013; López-Balderas et al., 2019; Yañez-Alvarez et al., 2012).

The highest seroprevalences of syphilis and active syphilis were among men and transgender people. It is possible that a high proportion of men attending the CEC are MSM, a group in which the HIV epidemic in Mexico is concentrated (Bautista-Arredondo et al., 2013). In the case of men, the high frequency and incidence of active syphilis could be related to sexual practices, unprotected anal sex, a large number of sexual partners, and/or casual relationships. The transgender population had different vulnerabilities, namely being sex workers, unfavorable economic conditions, and/or stigma and discrimination (Salas-Espinoza et al., 2017).

The highest frequency of active syphilis, as well as the highest incidence of syphilis, was found in 20–39-year age group. These data are consistent with the worldwide trend, which shows that the largest number of new STI cases occur in the young population (CDC., 2018). Thus, it is necessary to focus syphilis prevention and diagnosis programs in these groups. The presence of other STIs is a risk factor for the acquisition and transmission of *T. pallidum*, as shown with HIV and HBV but not HCV (whose main route of transmission is parenteral rather than sexual). PLWH may have a lowered immune response, which increases their susceptibility to other STIs, such as syphilis (Callegari et al., 2014). People with coinfection of HIV and syphilis have a higher risk of neurological complications, which may be more frequent, progress faster, and present atypical signs. These individuals may also have treatment failure or present serofast (individuals without syphilis but higher antibody titers) (Johns et al., 1987). On the other hand, syphilitic lesions increase the risk of acquiring and transmitting HIV; persons with syphilis have a fourfold greater risk of acquiring HIV (Galvin et al., 2004). This value is similar to the current study among people attending the CEC.

The higher prevalence and incidence of syphilis among PLWH may be due to individual risk perception and their social networks (Blair et al., 2019). From the onset of antiretroviral treatment, the number of deaths decreased, and life expectancy improved. Consequently, some PLWH continued engaging in risky sexual behaviors, such as unprotected anal sex, occasional sexual partners, concurrent sexual partners, serosorting, chemsex, transactional sex, methamphetamine use, group sex or contacting partners over the internet, and anonymous couples (Cohen et al., 2012, McNamara, et al., 2020).

A high percentage of people had persistent infection; that is, they maintained similar levels of antibodies during the study period. Failures in syphilis treatment have been documented from 3% among people without HIV to 17% among PLWH (Ghanem et al., 2007). Furthermore, among PLWH, the antibody titers take longer to decrease (serofast patients) in up to 25% of the population (Spagnuolo et al., 2018). The CEC prescribes the treatment for syphilis; however, it is unknown if people receive the treatment, as most of the participants are sent to their family medical clinic for follow-up. Only PLWH without social security are followed in the CEC.

There are few studies on the incidence of syphilis in Mexico. Among FSW, it was 4.31 cases per 100 py, and among their stable sexual partners, it was 3.64 cases per 100 py (Bazzi et al., 2015). In the current study, the VCT group had a similar incidence of 3.4 cases per 100 py. However, the highest incidence was among PLWH, with 16 cases per 100 py. The incidence of syphilis was higher than that detected in other countries, including Italy (1.94 per 100 py), Singapore (6.21 per 100 py), and Australia (5.47 per 100 py), all among PLWH (Ang et al., 2020; Goddard et al., 2019; Spagnuolo et al., 2018). This was similar to the very high incidence of syphilis reported in Argentina (14.9 cases per 100 py) (Bissio et al., 2017) In addition to the high incidence, there was a high percentage of syphilis reinfection. In other countries, this reinfection has been reported and appears to be a risk factor for reinfection, being asymptomatic, living with HIV, being a MSM, and failure in case follow-up (Kenyon et al., 2014).

In relation to limitations, the current study did not include sociodemographic, sexual behavior, and clinical information about participants; it was to difficult to know if all patients received the adequate treatment and follow-up because there is no epidemiological surveillance information about other clinics. The prevalence of active infection without clinical information may be overestimating the prevalence, especially among PLWH who present as serofast, and there was potential selection bias in the dynamic cohort due to the small number of cases with follow-up. Possibly, we are overestimating the prevalence of syphilis among PLWH because PLWH with social security have a better socioeconomic status, which makes them generally less vulnerable to various infections.

### Conclusions

Different vulnerable groups showed a high incidence and high prevalence of active syphilis and reinfections in the CEC. The results confirm that it is necessary to allocate more resources for the detection, treatment, and surveillance of syphilis cases, as suggested by the WHO, to control syphilis and eliminate congenital cases (WHO, 2016; WHO, 2018). Specific strategies need to be implemented, including continuous training for health personnel to identify, treat, and follow-up on syphilis cases, contact tracing of sexual partners, and sex education for vulnerable populations, among other prevention measures.
